# Systolic propulsion of the eyeballs in severe tricuspid regurgitation: a case series and review of the literature

**DOI:** 10.1515/med-2026-1395

**Published:** 2026-03-20

**Authors:** Hadi Beaini, Jessica Qiu, Corbin Foster, Anas Jawaid, E. Ashley Hardin, Maryjane Farr, Faris G. Araj

**Affiliations:** Department of Medicine, University of Texas Southwestern Medical Center, Division of Cardiology, Dallas, TX, USA

**Keywords:** physical exam, tricuspid regurgitation, jugular venous pressure, echocardiogram, prognosis, eyeball pulsation

## Abstract

**Objectives:**

Severe tricuspid regurgitation (TR) can be a phenotypic manifestation of an underlying disease process that is the primary driver of patient prognosis. Associated clinical signs such as small vein pulsations are similarly prognostic. Systolic propulsion of the eyeballs is an extreme manifestation of severe TR with only seven reported cases in the literature. We herein report two additional cases, review the available literature, and discuss prognostic implications of this phenomenon.

**Case presentation:**

Two patients with severe tricuspid regurgitation and systolic propulsion of the eyeballs are described in a comprehensive manner. Unlike previous reports, we include contemporary high-quality images and video of the phenomenon in addition to the most in-depth review of the literature on this subject to date.

**Conclusions:**

Systolic propulsion of the eyeballs is a manifestation of severe tricuspid regurgitation in the background of profound right ventricular dysfunction. We believe this is a rare clinical sign that carries ominous prognostic implications.

## Introduction

Severe tricuspid regurgitation results in impaired quality of life and increased morbidity due to the development of congestive right heart failure and low cardiac output [1]. In a contemporary randomized controlled trial evaluating the safety and effectiveness of tricuspid transcatheter edge-to-edge repair in symptomatic patients with severe tricuspid regurgitation, death from any cause or tricuspid valve surgery occurred in 10 % of patients at 1-year follow up [1].

The presence of certain overlooked clinical signs of severe tricuspid regurgitation such as small vein pulsations has similarly been associated with a poor prognosis. Taken together, this suggests that tricuspid regurgitation is a phenotypic manifestation of an underlying disease state that is the primary driver of outcome [2], 3].

Systolic propulsion or pulsation of the eyeballs is a rarely described clinical manifestation of severe tricuspid regurgitation with unknown prevalence [4], 5]. We here in describe two such cases and review the available literature on this phenomenon. We also suggest that the presence of systolic pulsation of the eyeballs as an extreme manifestation of severe tricuspid regurgitation is prognostically relevant.

## Case presentations

### Case 1

**Figure 1:**
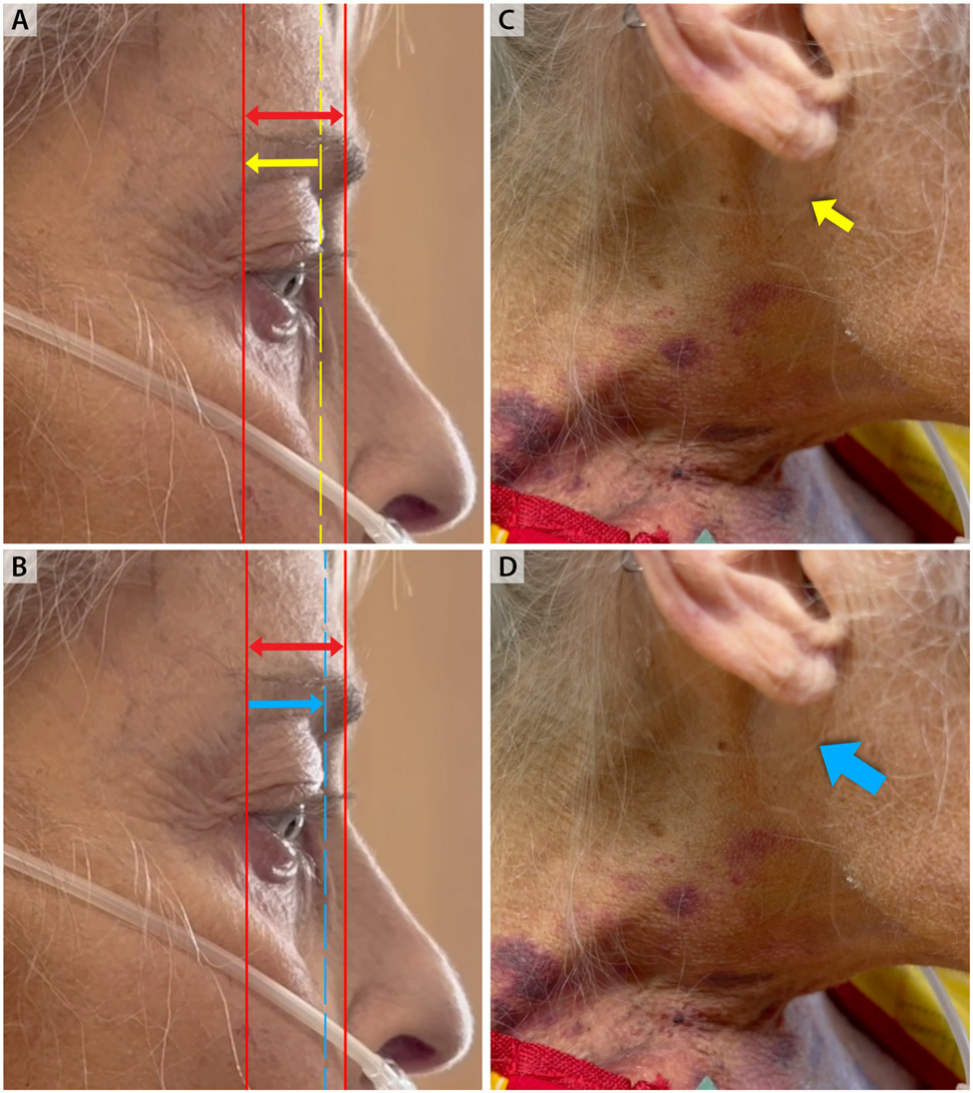
**Systolic pulsation of the eyeball and severe tricuspid regurgitation. Eyes open at 90°.** Solid red lines in the upper and lower panels (A,B) represent the borders of the angle of the eye and nasal bridge. In yellow, retraction of the eyeball during diastole relative to predefined borders (panel A) and nadir of jugular venous y- descent (small yellow, arrow panel C). In blue, maximal excursion of the eyeball during systole relative to free defined borders (panel B) and peak jugular venous V wave (big blue arrow, panel D).

**Figure 2:**
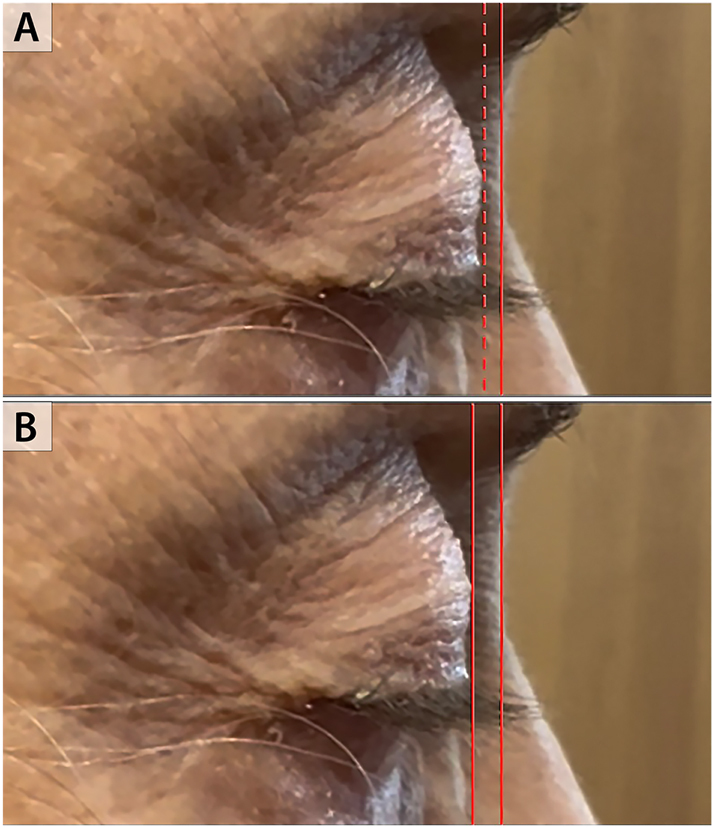
**Systolic pulsation of the eyeball. Eyes closed at 90°.** Solid red line at the nasal bridge represents a common reference point for both upper and lower panels. Dotted red line in showing maximal excursion of the eyeball during systole (panel A). Retraction of the eyeball back to baseline during diastole (panel B).

The patient is a 65-year-old female with a history of Hodgkin’s lymphoma and mantle field radiation 50 years prior to presentation. As a result of radiation therapy, she developed radiation heart disease necessitating aortic valve replacement approximately 14 years earlier, in addition to radiation-induced coronary artery disease, and restrictive cardiomyopathy. Over the years she has had several hospitalizations for acute chronic decompensated heart failure with right-sided symptoms including increased abdominal girth, lower extremity edema and recurrent pleural effusions despite high-dose oral diuretics. Within the same period, she was also noted to have progression in the degree of her tricuspid valve regurgitation from moderate regurgitation one year prior to presentation to severe tricuspid regurgitation at the time of presentation. Hypotension necessitated midodrine to maintain blood pressure. There was no history of head trauma or surgery, and the patient reported no visual disturbance. Physical examination performed with the patient sitting upright was notable for shortness of breath at rest with hypoxia and O2 saturation of 92 %. Pulse was 80 bpm. Jugular venous pressure was markedly elevated above the angle of the jaw. The jugular venous waveform exhibited a monophasic peak and brisk trough corresponding to the prominent V-wave and Y-descent, respectively. There were no bruits heard on auscultation of the neck. Slight exophthalmos and pulse-synchronous systolic pulsation of the eyeballs was noted (Figures 1 and 2; Videos 1-3) Heart sounds were regular, and a systolic murmur was present in addition to an S3. Breath sounds over the right posterior lung field were diminished, and mild abdominal distention with a fluid wave was noted. There was significant bilateral pitting edema of the lower extremities.

**Figure 3:**
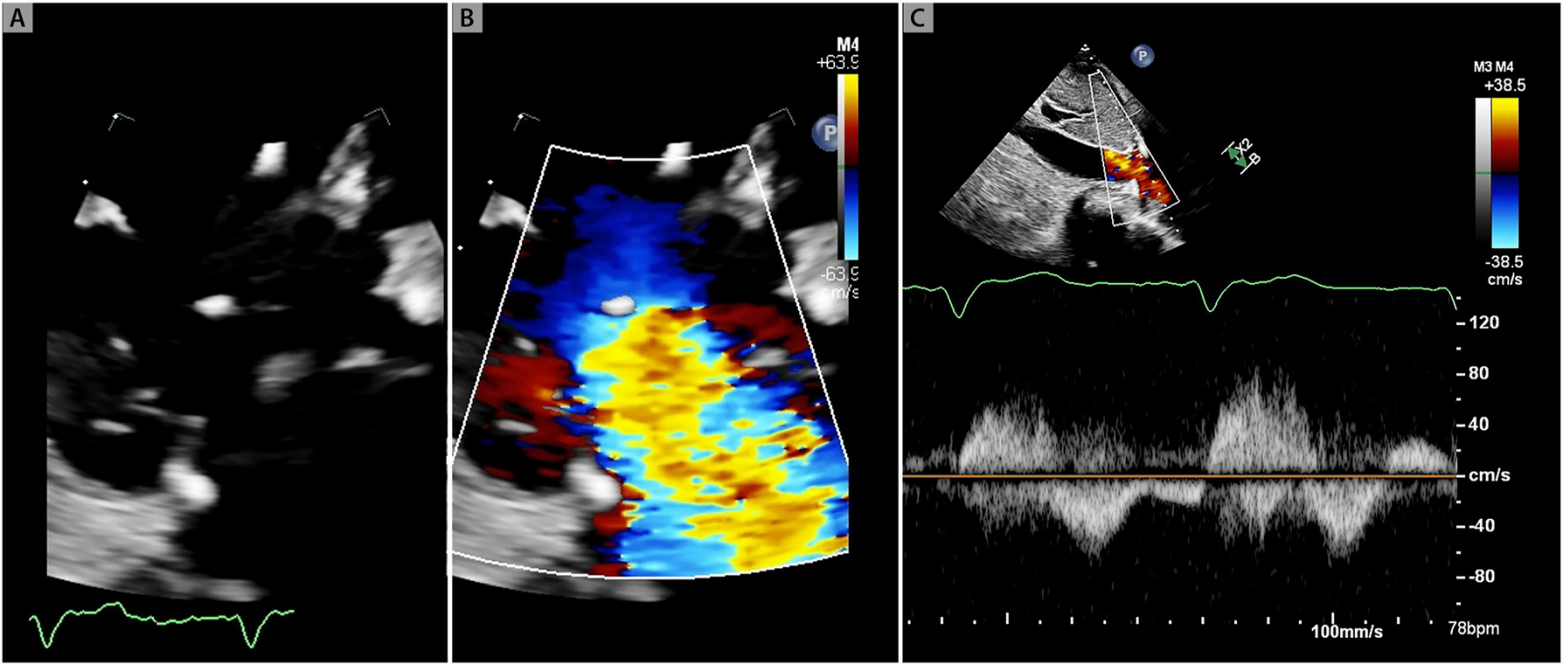
**Echocardiogram images showing severe TR.** Right ventricular inflow view. Malcoaptation of the tricuspid valve leaflets (panel A). Severe tricuspid regurgitation by color Doppler (panel B). Systolic flow reversal in the hepatic vein (panel C).

**Figure 4:**
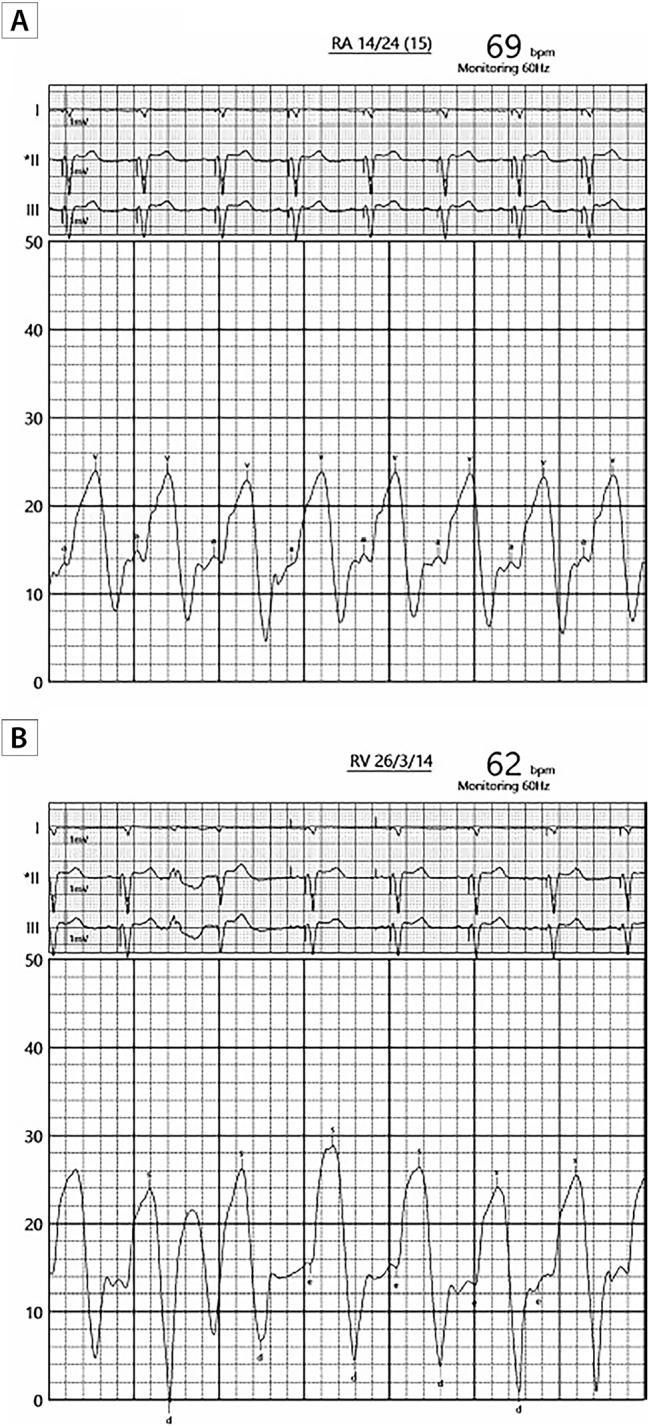
**Invasive hemodynamic tracings of severe tricuspid regurgitation.** Ventricularization of the right atrial pressure tracing is noted. Peak V wave of 24 mmHg (panel A). Right ventricular pressure tracing appears identical to the right atrial pressure tracing. Peak right ventricular systolic pressure is 26 mmHg approximating the peak V wave pressure in the right atrial tracing. (Panel B).

She was subsequently admitted to the hospital for evaluation and treatment of acute decompensated heart failure and was initiated intravenous diuretic therapy. Investigations that were performed included an echocardiogram that showed moderately depressed right ventricular systolic function with associated wide-open severe tricuspid valve regurgitation with poorly coapting tricuspid valve leaflets and systolic flow reversal noted in the hepatic veins (Figure 3) The bioprosthetic aortic valve function appeared normal. LV systolic function was normal with an LVEF of 55 to 60 %. Simultaneous right and left heart cardiac catheterization showed elevated and equalized diastolic pressures with respiratory concordance between the right and left sided chambers, consistent with restrictive cardiomyopathy rather than constrictive pericarditis. There was no significant pulmonary hypertension with a PA pressure of 26/14 (mean of 20 mmHg). Right atrial and right ventricular waveforms were nearly identical, suggesting ventricularization of the right atrial pressure tracing consistent with severe tricuspid regurgitation (Figure 4).

Cardiac index was low (less than 2.2 L/min/m^2^). Transesophageal echo was notable for preserved LV systolic function LVEF 55 % right ventricular systolic function was depressed with a dilated right ventricle tricuspid valve was with thickened leaflets with poor leaflet coaptation and wide-open severe central tricuspid regurgitation. Given the above data it was felt that the severe tricuspid regurgitation in the background of radiation valve and heart disease is contributing most to her symptoms. Inotrope therapy was added.

**Figure 5:**
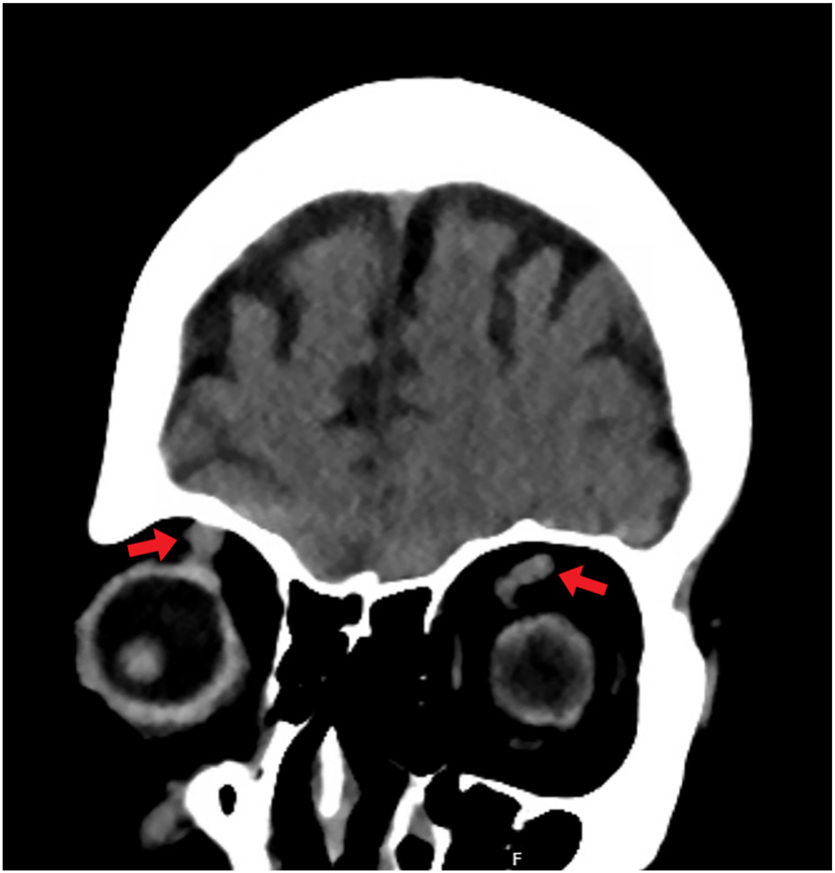
**Computed tomography of the head. Coronal en face view.** Enlargement of the superior ophthalmic veins is noted as a consequence of severe tricuspid regurgitation and markedly elevated venous pressure.

Due to the presence of radiation-induced heart and lung disease and heart failure with preserved ejection fraction, she was not felt to be a candidate for advanced heart failure therapies. A multidisciplinary valve team felt she was too high risk for surgical valve replacement and anatomically the tricuspid valve was not suitable for tricuspid edge-to-edge repair due to too wide of a coaptation gap. Due to episodes of altered mental status, a CT of the head was performed, which showed no acute stroke; however, enlargement of the orbital veins was incidentally noted (Figure 5).

Hypotension refractory to oral midodrine ensued and the patient was initiated on vasopressors. Empiric antibiotics were started in addition to stress dose steroids. She subsequently developed worsening renal function and anuric renal failure requiring initiation of renal replacement therapy. There was progression of shock, escalation in vasopressor requirements, worsening mental status and decreased alertness level, and inability to move volume due to hypotension. The family members decided to transition to comfort care.

### Case 2

Patient is a 44-year-old man with severe nonischemic cardiomyopathy who was admitted with acute on chronic decompensated systolic heart failure. His symptoms included worsening exertional dyspnea and lower extremity edema. N-terminal proBNP level was 3500 (normal less than 300 pg/mL). Over the years he has had multiple hospitalizations for acute decompensated systolic heart failure and was in New York Heart Association class III-IV. There was no history of head trauma or surgery, and the patient reported no visual disturbance.

**Figure 6:**
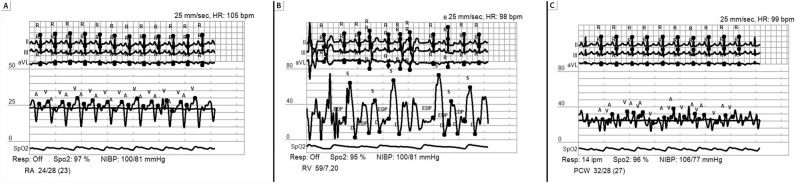
**Hemodynamics of severe heart failure.** Invasive hemodynamic tracings 2 years prior to presentation suggestive of underlying severe cardiomyopathy with evidence of markedly elevated intracardiac filling pressures. Right atrial, right ventricle, pulmonary capitally wedge pressure tracings in panels A, B, C, respectively.

**Figure 7:**
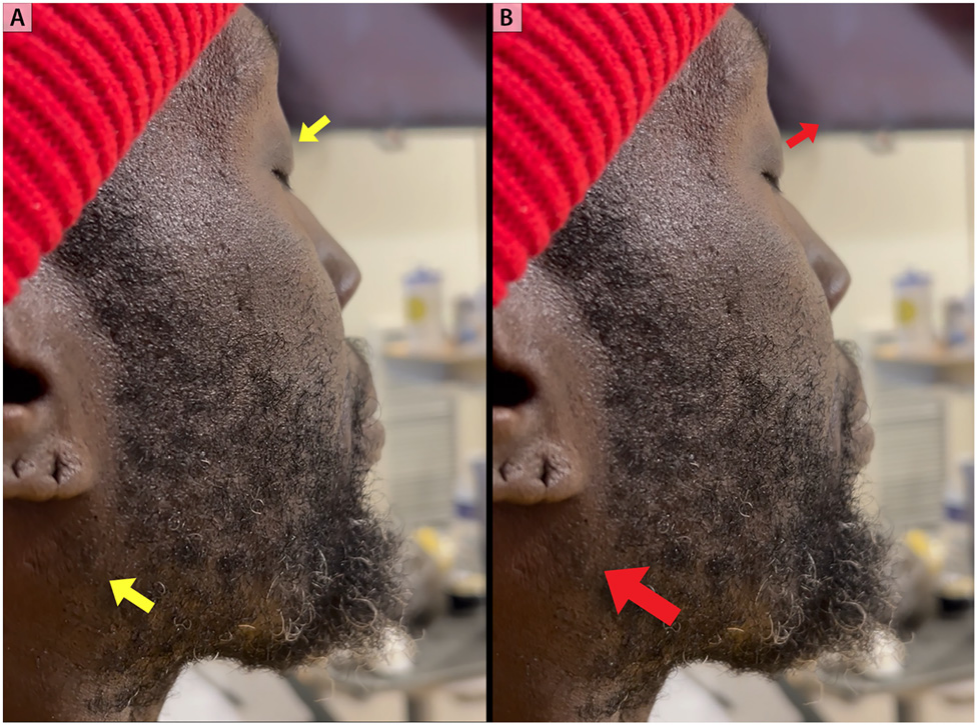
**Systolic pulsation of the eyeball. Eyes closed at 90°.** Retraction of the eyeball back to baseline during diastole. Big yellow arrow points to jugular venous pressure at its nadir (panel A). Maximal excursion of the eyeball during systole. Red arrow points to jugular venous pressure at its peak V-wave (panel B).

**Figure 8:**
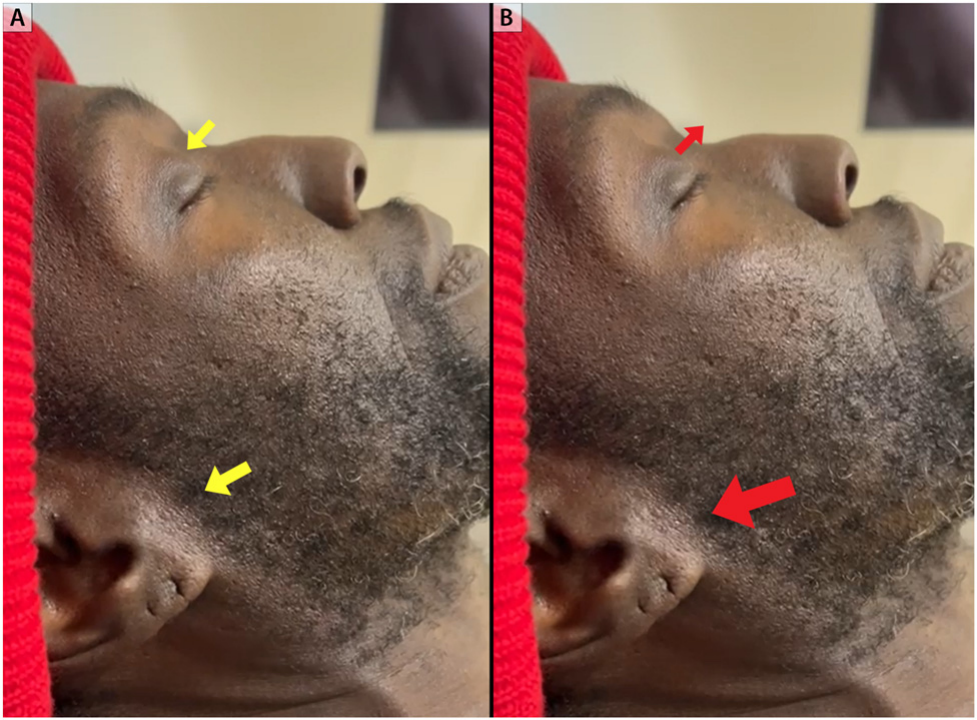
**Systolic pulsation of the eyeball. Eyes closed at 45°.** Retraction of the eyeball back to baseline during diastole. Big yellow arrow points to jugular venous pressure at its nadir (panel A). Maximal excursion of the eyeball during systole. Bigger red arrow points to jugular venous pressure at its peak V-wave (panel B).

**Figure 9:**
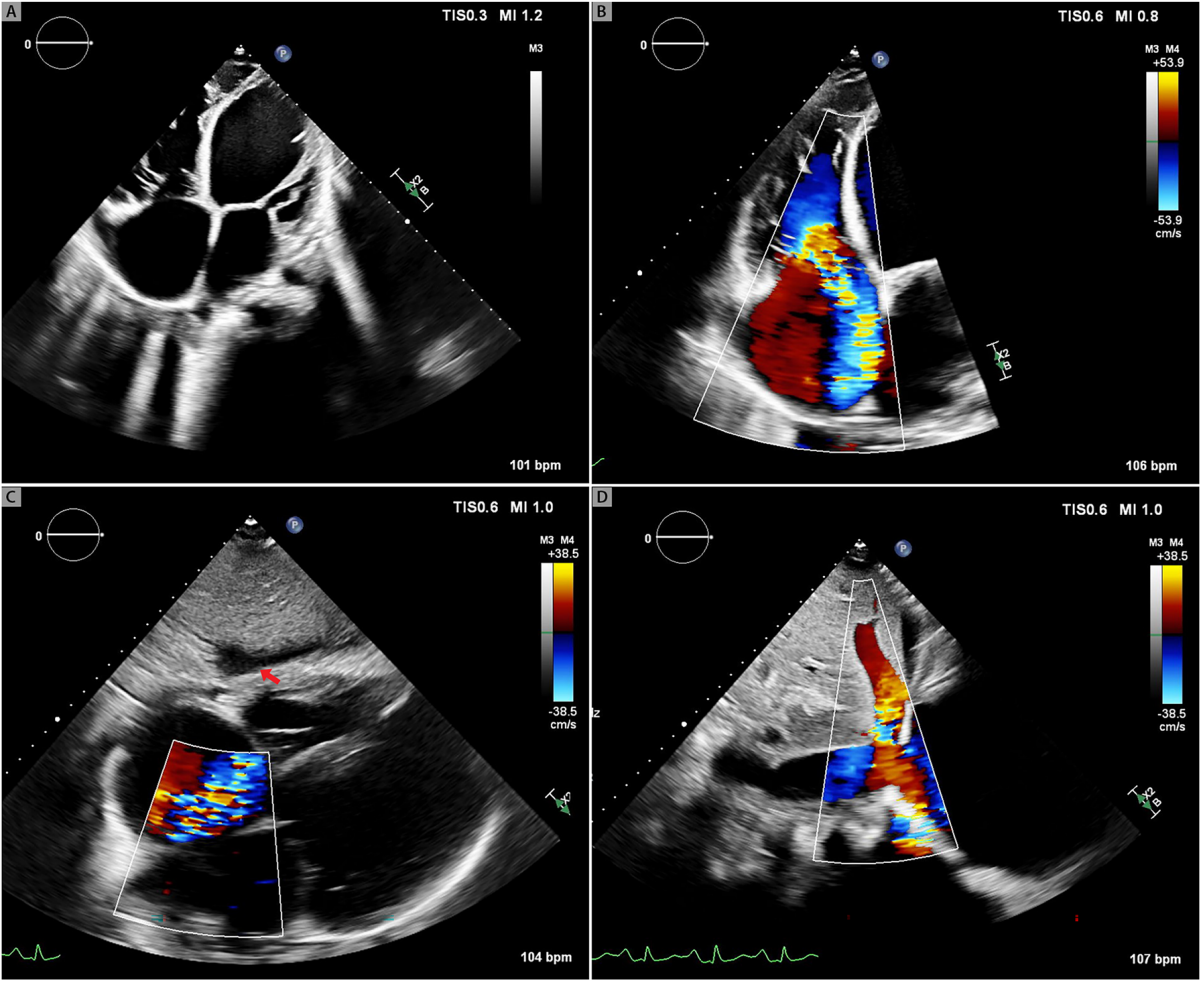
**Echocardiogram of severe heart failure and severe tricuspid regurgitation.** 2D image showing four-chamber enlargement and severe biventricular dysfunction. (Panel A). Severe tricuspid regurgitation (panel B). Perihepatic fluid (red arrow, panel C). Systolic flow reversal in the hepatic vein by color Doppler (panel D).

Echocardiogram two years prior to presentation showed a severely dilated left ventricle measuring 7.4 cm, severely reduced biventricular systolic function with at least moderate mitral regurgitation and severe tricuspid regurgitation with systolic flow reversal in hepatic veins. Right heart catheterization two years prior to presentation was consistent with severe and advanced heart failure with a mean right atrial pressure of 23 mmHg, PA pressure of 57/31 mmHg (mean of 49 mmHg), pulmonary capillary wedge pressure of 27 mmHg, and a low cardiac index of 1.1 L/min/m^2^ (Figure 6). Physical examination was notable for marked elevation in jugular venous pressure with the jugular venous waveform exhibiting a monophasic peak and brisk trough corresponding to the prominent V-wave and Y-descent, respectively. Pulse synchronous systolic propulsion of the eyeballs was also present (Figures 7 and 8; Videos 4,5). There were no bruits heard on auscultation of the neck. Cardiac auscultation was notable for holosystolic murmur at the left lower sternal border and at the apex. S3 was present. Bilateral lower extremity edema was present. Echocardiogram showed severely depressed LV systolic function with LVEF of 5 %, with low echocardiographic stroke-volume estimate, in addition to severe mitral and tricuspid valve regurgitation (Figure 9).

Intravenous diuretic and inotrope therapy were initiated, with improvement in his jugular venous distention. The patient was not a candidate for advanced heart failure therapies due to medication adherence concerns, and he did not want to go home on palliative inotrope therapy. Unfortunately, he was readmitted less than 1 month later in cardiogenic shock with systolic blood pressure 80 mmHg and lactic acid level of 3.5 mmol/L. This culminated in multisystem organ failure and death.

### Informed consent

Written informed consent for publication was obtained from the subjects.

## Discussion

In this case series we shed new light on a rarely described physical exam finding of severe tricuspid regurgitation: systolic propulsion of the eyeballs.

Our report is unique for several reasons, the first of which is that it adds to the already scant data regarding this physical exam finding and its clinical significance. To our knowledge there are only seven other cases in the literature describing systolic pulsation of the eyeballs in the setting of severe tricuspid regurgitation. Second, we provide the most comprehensive multimedia documentation available in the literature to date, highlighting this phenomenon and including extensive hemodynamic and echocardiographic data. Third, this is the most comprehensive review of the literature regarding this physical exam finding to date. Lastly, unlike the pre-existing cases, we have shown the association of this extreme physical exam finding with poor patient prognosis and therefore reinforcing the relevance of the bedside cardiovascular physical exam.

### The physical examination in severe tricuspid regurgitation

The classic triad of severe tricuspid regurgitation is the Rivero-Carvallo sign, prominent jugular venous V waves, and pulsatile liver [5]. However, less well known far-reaching phenomena of severe tricuspid regurgitation have also been described, including pulsation of the veins of the forearms, chest wall, face, varicoceles, and varicose veins [2]. Systolic propulsion of the eyeballs as a sign of severe tricuspid regurgitation has mostly been overlooked as evidenced by a contemporary review of ocular manifestations of systemic and cardiovascular abnormalities [6].

The clinical hallmark of severe tricuspid regurgitation is a monomorphic jugular venous pulsation also known as Lancisi’s sign [7]. The normal three peaks and two troughs of the venous waveform are replaced by a fused C-V wave during ventricular systole that is often palpable, followed by an accentuated Y descent [7]. In our experience, this has been often mistaken for an arterial pulse even by upper internal medicine residents.

In 1970 Drs Earnest and Hurst were first to describe the phenomenon of systolic propulsion of the eyeballs due to severe tricuspid regurgitation [4]. This observation was published in the American Journal of Cardiology [4]. Nearly half a century later, this phenomenon has rarely been reported, and to the best of our knowledge, video documentation of this phenomenon does not exist on the internet or in any Supplementary material related to any publication on the subject.

### Pathophysiology of systolic propulsion of the eyeballs in severe tricuspid regurgitation

Systolic propulsion of the eyeballs is an extreme manifestation of tricuspid regurgitation and requires the presence of both severe venous hypertension and severe tricuspid regurgitation [2], 4], 8]. Venous hypertension alone is insufficient to elicit this exam finding [4], 8]. When normal individuals were asked to stand upside down for 5 min to acutely increase the venous pressure in the head, systolic propulsion of the eyeballs was not seen despite all subjects showing cervical venous distention, facial plethora, and varying degrees of exophthalmos [4].

The venous plexus of the eyes and the retro-orbital spaces drain into the cavernous sinus and jugular venous system. When the central venous pressure is increased, this can exert influence on the orbital contents [4]. Systolic propulsion of the eyeballs depends on transmission of RV systolic impulse to the cephalic venous system. The superior and inferior ophthalmic veins are valveless, allowing for transmission of the jugular CV wave via the cavernous sinus, resulting in displacement of the eyeballs during systole [4], 8]. This is supported by the finding in our case series involving patient 1, in which computed tomography of the brain incidentally showed enlargement of the orbital veins (Figure 5).

The CV wave pressure required to cause systolic propulsion of the eyeballs is not well defined. In the original description venous pressure was measured in the medial antecubital vein against a saline column and venous pressure ranged from 145–380 mm of saline (10.6–28 mmHg) in the cohort of fifteen patients. Only three patients were noted to have systolic propulsion of the eyeballs, but it is not known what their individual measured pressures were [4]. The other descriptions in the literature did not provide invasive hemodynamics. The available invasive measurements in our case series are consistent with marked elevations in right atrial pressure with peak V-wave measuring 26–28 mmHg.

To put this in the context of a contemporary randomized controlled trial evaluating the safety and effectiveness of tricuspid transcatheter edge-to-edge repair in symptomatic patients with severe tricuspid regurgitation, the mean CVP was 11–12 mmHg, with nearly 60 % in NYHA class III–IV and 25 % with one hospitalization for HF within 1 year before enrollment [1].

### Physical examination of systolic propulsion of the eyeballs

It has been suggested that an ocular assessment should accompany the cardiovascular examination as ocular manifestations may indicate underlying cardiovascular pathology [6]. Heart failure has been associated with exophthalmos with stare and increased intraocular pressure, both of which can improve with decongestion [4], 6].

Upon exam, the phenomenon involves both eyes and is more likely to be noticed with the patient seated at 45° and looking straight across paying attention to the curve of the eyes from either side [8], 9]. A detailed description is available in Table 1. In our case series, this phenomenon was seen in the upright position in both patients. There does not appear to be a particular height of the jugular venous column on exam to predict whether this exam finding is present. For example, in the report by Allen and Naylor despite the presence of pulsating earlobes in the upright position (90°), systolic pulsation of the eyeballs was only seen at 45° [8].

**Table 1:** Literature describing systolic pulsation of the eyeball in severe tricuspid regurgitation.

Author	Number of patients	Etiology of TR	Additional signs & symptoms	Exam	Invasive pressure measurement	Vision changes	Outcome	Long-term follow-up
Naylor	1	Ischemic heart disease with predominantly right-sided failure	JVD, systolic murmur ↑ with inspiration, edema, pulsatile liver	Reclined at 45°	No	No	Resolved with diuresis	No
Allen et al.	2	1) Mild pulmonary HTN with RV overload from tumor	1) Pansystolic murmur LLSB, JVD to jaw, pulsatile liver	1) Reclined at 45°	No	1) No	1) Death (likely malignancy)	No
		2) Mild cardiomyopathy / idiopathic myxomatous degeneration of the valve	2) Ankle edema, DOE, JVD with “CV wave” to earlobe,2/6 systolic murmur LLSB, pulsatile liver	2) Reclined at 45°, not seen at higher degrees	No	2) No	2) Resolved with diuresis	
Chen et al.	1	Ischemic heart disease and atrial fibrillation	Orthopnea, ankle edema, JVD at 90° (indiscernible waveform), tense ascites, no murmur	Seen at all angles, including 90°	No	No (optic discs normal)	Resolved with diuretics	No
Earnest et al.	3	Heart failure (not specified if preserved or reduced EF)	JVD, edema, pulsatile liver	Reclined at 30°, not seen at all angles	Measured in the antecubital vein against a saline column (10.6–28 mmHg)	No; possible asymptomatic increased intra-ocular pressure	Not specified	No

JVD, jugular venous distension; LLSB, left lower sternal border; DOE, dyspnea on exertion; EF, Ejection fraction.

Eyeball pulsation in this context is dynamic and tracks with the state of congestion. The pulsation of the eyeballs has been noted to improve or disappear when jugular venous pressure is lowered sufficiently [8], [9], [10].

Systolic propulsion of the eyeballs is often not present in the absence of other signs of tricuspid regurgitation (Table). However, in the absence of a significant elevation of the jugular venous pressure systolic propulsion of the eyeballs has not been described [8]. Rarely has eyeball pulsation been the only clinical finding on presentation to suggest a diagnosis of tricuspid regurgitation [10]. In this circumstance, however, the authors note that the jugular venous pressure was elevated to a point whereby the pulsation of the waveform was indiscernible even in the upright position, no murmur was documented, yet the patient had edema and ascites [10].

### Differential diagnosis

In addition to severe tricuspid regurgitation, systolic propulsion of the eyeball has been described in other conditions. The differential diagnosis of a pulse-synchronous pulsation of the eyeball includes orbital roof fractures, congenital skull-base defects, encephaloceles or meningoceles, neurosurgical procedures, neurofibromatosis type 1 (sphenoid wing dysplasia), and vascular malformations such as carotid-cavernous fistulas, arteriovenous malformations, and arachnoid cysts [11]. Unlike systolic propulsion of the eyeball in severe tricuspid regurgitation, which is bilateral, painless, and not associated with changes in vision [4], [5], [6, [8], [9], [10], in these alternate conditions the finding is unilateral (i.e., involves only one eye and is usually ipsilateral to the underlying pathologic condition). A bruit may be present, there may be pain and vision loss, there is no variation in eye movement with changes in posture or position of the trunk, and other stigmata of tricuspid regurgitation are absent [4], 8], 10]. Non-pulsatile orbital pain in the setting of acute decompensated heart failure and orbital varix mimicking intraorbital lymphoma has been reported [12]. This symptom improves with diuresis and may represent a manifestation of volume overload.

### Prognostic implications

It has been previously shown that pulsation of small veins should always be considered abnormal [2], 3], 13]. Not only is it abnormal, but in the context of severe tricuspid regurgitation, the presence of small vein pulsation is also of prognostic significance, portending a worse outcome [2], 3]. Our case series further highlights the poor prognosis associated with systolic propulsion of the eyeballs in the setting of severe tricuspid regurgitation. In this context, tricuspid regurgitation should be approached as a symptom of an underlying pathology that may not be altered by addressing the valve lesion alone.

There remain uncertainties regarding patient prognosis and this physical exam finding. For example, in our two patients, systolic propulsion of the eyeballs was seen at 90° and both patients died. We do not know whether inducibility of this exam finding carries the same association with prognosis. This is to say that if systolic propulsion of the eyeballs is not seen at 90°, it may be elicited at 45°, or at either angle by applying pressure over the abdomen, i.e., the abdominojugular reflux test. Does resolution of this physical exam finding as the jugular venous pressure is lowered favor a better long-term outcome for the patient? In case two, despite improvement in jugular venous pressure with IV diuretic and inotrope therapy, the patient presented again less than a month later in cardiogenic shock and died. We acknowledge that this is a rare phenomenon. More studies are needed to explore the topic and validate the prognostic value of the sign.

## Conclusions

Systolic propulsion or pulsation of the eyeballs is a rarely described clinical manifestation of severe tricuspid regurgitation and is of prognostic importance. However, technology and time constraints have overshadowed clinical bedside examination skills, such that the physical examination is no longer performed proficiently despite literature showing that certain bedside examination findings are prognostically relevant [2], 14], 15].

Separate from the presence of a third heart sound and elevated jugular venous pressure, overlooked clinical signs of severe tricuspid regurgitation, such as small vein pulsations, have also been associated with a poor prognosis. We now add to this literature by describing the prognostic implications of systolic pulsation of the eyeballs. This clinical phenomenon should be considered a marker of profound right ventricular dysfunction, warranting consideration for advanced heart failure therapies rather than tricuspid valve intervention. Taken together, this suggests that tricuspid regurgitation is a phenotypic manifestation of an underlying disease state that is the primary driver of outcome, and treatment focus should not be shifted away from the ventricle in favor of the valve [2], 3].

## Supplementary Material

Supplementary Material

Supplementary Material
