# Supplementary material for: Systolic propulsion of the eyeballs in severe tricuspid regurgitation: a case series and review of the literature
Source: Open Med (Wars). 2026 Mar 20;21(1):20261395. doi: 10.1515/med-2026-1395 (PMC13001992; doi:10.1515/med-2026-1395)
Supplement: Supplementary file 1 — Supplementary Material [file j_med-2026-1395_suppl_001.docx]

**Supplementary video link:**

**P1 V1.mp4 -** <https://365utsouthwestern-my.sharepoint.com/:v:/g/personal/hadi_beaini_utsouthwestern_edu/ET9-YqUjEDFHtcPoM_PQBkIB6yfHXRym_oJiJe9y4xH8AA?e=vnKUZv&referrer=Outlook.Desktop&referrerScenario=email-linkwithoutembed>

**P2 V1.MOV -** <https://365utsouthwestern-my.sharepoint.com/:v:/g/personal/hadi_beaini_utsouthwestern_edu/ER-Fi-4G03lAs8DrYf56B7IBxy4jgpmeJTaTC4YUVBFg1A?e=lUvilC&referrer=Outlook.Desktop&referrerScenario=email-linkwithoutembed>

**P1 V2.MOV -** <https://365utsouthwestern-my.sharepoint.com/:v:/g/personal/hadi_beaini_utsouthwestern_edu/Ea_797yQWJBLiSzxuTUzryYBYRYOD1o_sevEo0rZ7gyCog?e=jFDyKl&referrer=Outlook.Desktop&referrerScenario=email-linkwithoutembed>

**P2 V2.mp4 -** <https://365utsouthwestern-my.sharepoint.com/:v:/g/personal/hadi_beaini_utsouthwestern_edu/ERZFjnz5RkRDhu-huJrP4yoBBbsKD7Q2lTEJPUvTvk5jxA?e=7aJXTQ&referrer=Outlook.Desktop&referrerScenario=email-linkwithoutembed>

**P1 V3.MOV -** <https://365utsouthwestern-my.sharepoint.com/:v:/g/personal/hadi_beaini_utsouthwestern_edu/EfxbUJUUk61KkMfmJcSNDbcBofLicF5awpw1NRfi7Hf-xw?e=ZNMPFo&referrer=Outlook.Desktop&referrerScenario=email-linkwithoutembed>
