# Supplementary material for: Systolic propulsion of the eyeballs in severe tricuspid regurgitation: a case series and review of the literature
Source: Open Med (Wars). 2026 Mar 20;21(1):20261395. doi: 10.1515/med-2026-1395 (PMC13001992; doi:10.1515/med-2026-1395)
Supplement: Supplementary file 2 — Supplementary Material [file j_med-2026-1395_suppl_002.docx]

**Video Legends**

**Patient 1**

**Video 1.** (combined eyeball + JVP) Pulse synchronous systolic pulsation of the eyeball. Eyes open at 90 degrees. Eyeball pulsation on the left and jugular venous pulsation of severe TR on the right.

**Video 2.** (Eyeball alone- side view). Pulse synchronous systolic pulsation of the eyeball. Eyes open at 90 degrees.

**Video 3.** (Eyeball eye closed upper shot) Pulse synchronous systolic pulsation of the eyeball. Eyes closed at 90 degrees.

**Patient 2**

**Video 1.** (90 degrees) Pulse synchronous systolic pulsation of the eyeball. Eyes closed at 90 degrees. Marked elevation of the jugular venous pressure and jugular venous V wave is noted.

**Video 2.** (45 degrees) Pulse synchronous systolic pulsation of the eyeball. Eyes closed at 45 degrees. Marked elevation of the jugular venous pressure and jugular venous V wave is noted.
